# Supplementary material for: Interdependent Regulation of Polycystin Expression Influences Starvation-Induced Autophagy and Cell Death
Source: Int J Mol Sci. 2021 Dec 16;22(24):13511. doi: 10.3390/ijms222413511 (PMC8706473; doi:10.3390/ijms222413511)
Supplement: Supplementary file 1 [file ijms-22-13511-s001.zip › Supplementary Figure Legends_rev.pdf]

Supplementary Figure Legends

Figure S1: Illustration, genomic validation and characterization of mIMCDs. (**A**) Diagram illustration of mouse Pkd1 genome covering the exon 2 and exon 3. The confirmed deletion of DNA fragment is indicated by dash line. (**B**) DNA sequence alignment of the PCR products using primers the Forward primer and Reverse primer. (**C**) PCR products were shown in agarose gel. (**D**) The trace of DNA sequence. (**E**) Representative images of WT, PC1KO and PC2KO mIMCDs. Scale bar = 100 µm. (**F**) Proliferation rate, plotted as the increase in number of cells over time, of WT, PC1KO and PC2KO mIMCDs. (**G**) ATP/ADP ratio in WT, PC1KO and PC2KO mIMCDs (N = 5).

Figure S2: Basal and starvation-induced mTOR and AMPK are not altered in PC1KO compared to WT mIMCDs. (**A**) Western blotting of phosphorylated AMPK, total AMPK, total ULK1 and ULK1 phosphorylated at various sites (S757 as mTOR phosphorylated site and S555 and S317 as AMPK phosphorylated site) in lysates from WT and PC1KO mIMCDs incubated in full medium (0 h). Right: Representative Western blot; Left: Quantification of phosphorylated protein levels over total levels or ULK1 or AMPK over Actin (N = 3). (**B**) Western blotting of phosphorylated AMPK, total AMPK, total ULK1 and ULK1 phosphorylated in lysates from WT and PC1KO mIMCDs subjected to 48 h of nutrient starvation (48 h). Right: Representative Western blot; Left: Quantification of phosphorylated protein levels over total levels or ULK1 or AMPK over Actin (N = 3).

Figure S3: Normal autophagic response following 3 h of nutrient starvation in WT and PC1KO mIMCDs, but a reduction in PC2KO mIMCDs. (**A**) LC3-II levels were analyzed in protein lysates from cells subjected to 0 h or 3 h of nutrient starvation, in the presence of DMSO (−Baf A1) or Bafilomycin A1 (100 nM; +Baf A1). Left: Representative Western blot; Right: analysis of LC3-II levels over Actin in the conditions with Baf A1. Paired observations of each independent experiment are represented by the same symbol (N = 3). (**B**) GFP-LC3 punctae analysis in WT and PC1KO mIMCDs following 3 h of starvation. Left: Representative GFP, DAPI and merged images (scale bar = 20 µm); Right: quantification of the number of GFP-LC3 punctae per cell following 0 h and 3 h of starvation. Paired observations of each independent experiment are represented by the same symbol (N = 6). * $p < 0.05$, representing significant effect of the treatment (starvation) as assessed by Two-Way ANOVA

Figure S4: Proximal tubular cell lines derived from ADPKD patients. Western-blot analysis the annotated proteins in proximal tubular epithelial cells (PTECs) from young 3 healthy individuals (Control1, 2 and 3) and ADPKD patients (ADPKD 1,2 and 3). For each individual, 3 cell lines were analyzed (a and b). Human Embryonic Kidney (HEK) cell lysate and lysate of human renal tissue (Kidney) were used as controls. Cells were incubated for 10 days at 37°C to induced SV40 Large T antigen (SV40 LT) breakdown. P-Glycoprotein (PgP) and Epithelial Cell Adhesion Molecule (EpCAM) are epithelial markers and Aquaporin 1 (AQP1) a PTEC marker.
