# Supplementary figures and images for: Interdependent Regulation of Polycystin Expression Influences Starvation-Induced Autophagy and Cell Death
Source: Int J Mol Sci. 2021 Dec 16;22(24):13511. doi: 10.3390/ijms222413511 (PMC8706473; doi:10.3390/ijms222413511)

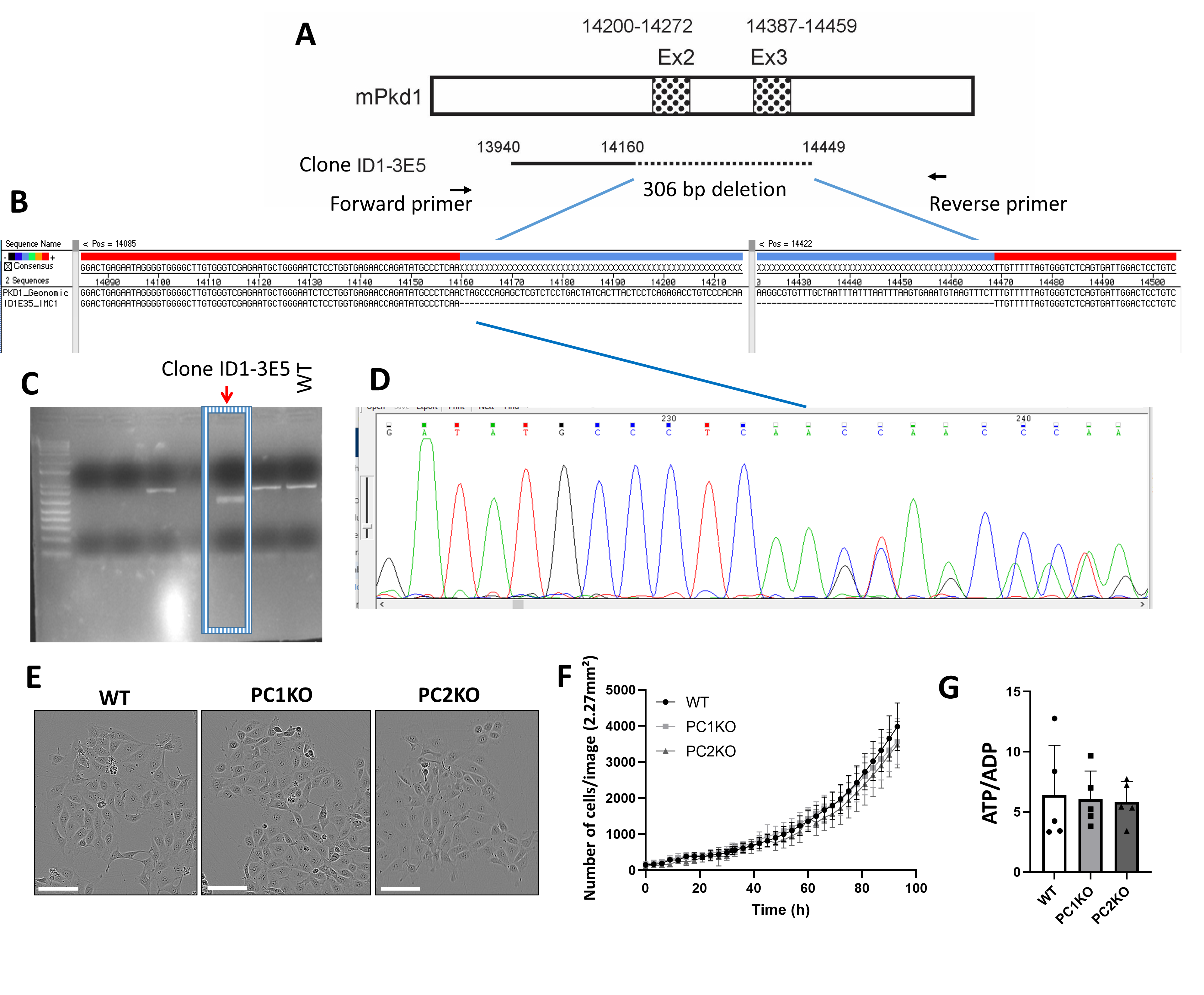

Supplement: Supplementary file 1 [file ijms-22-13511-s001.zip › New Fig. S1_v3.tif]

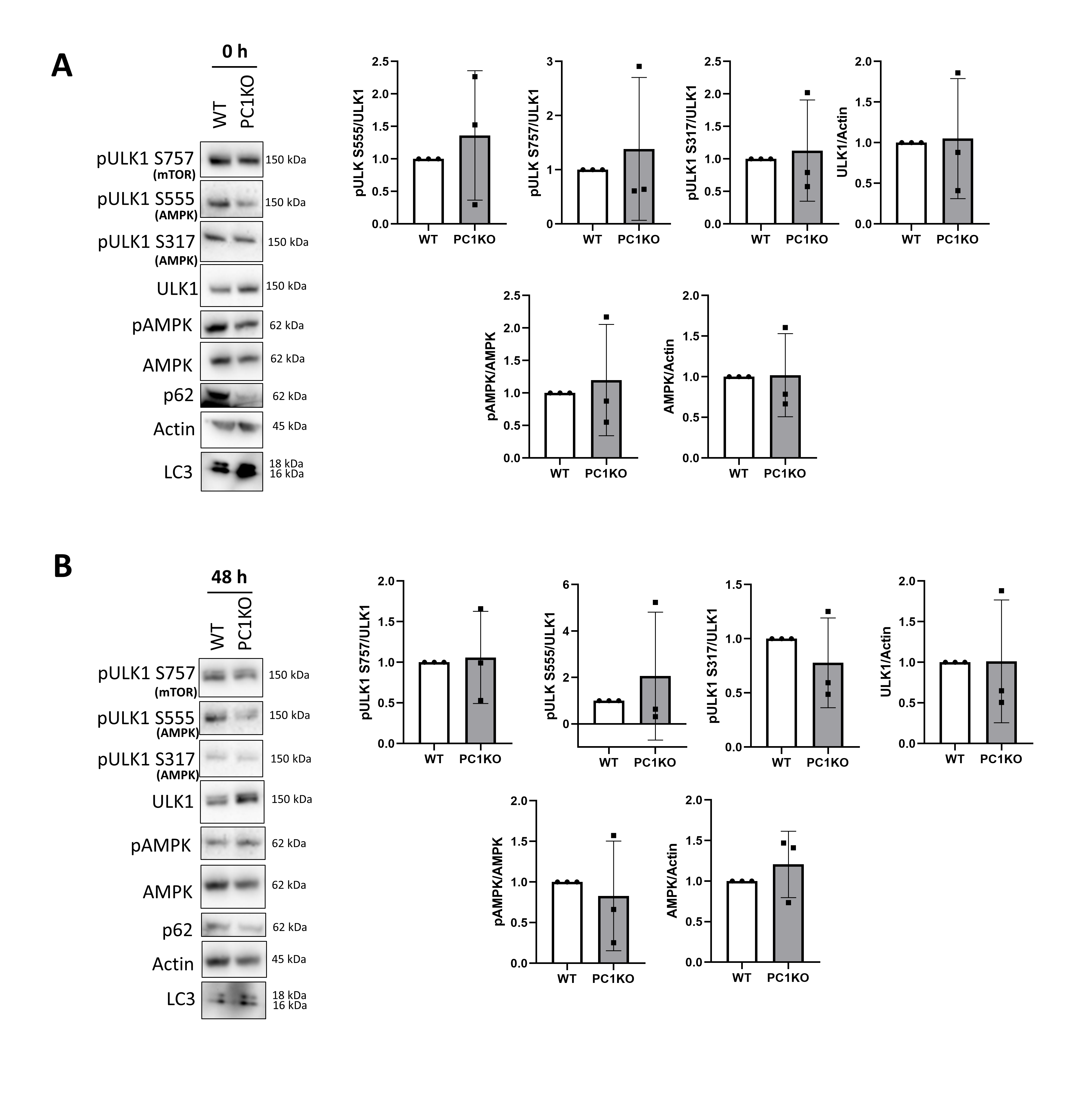

Supplement: Supplementary file 1 [file ijms-22-13511-s001.zip › New Fig. S2.tif]

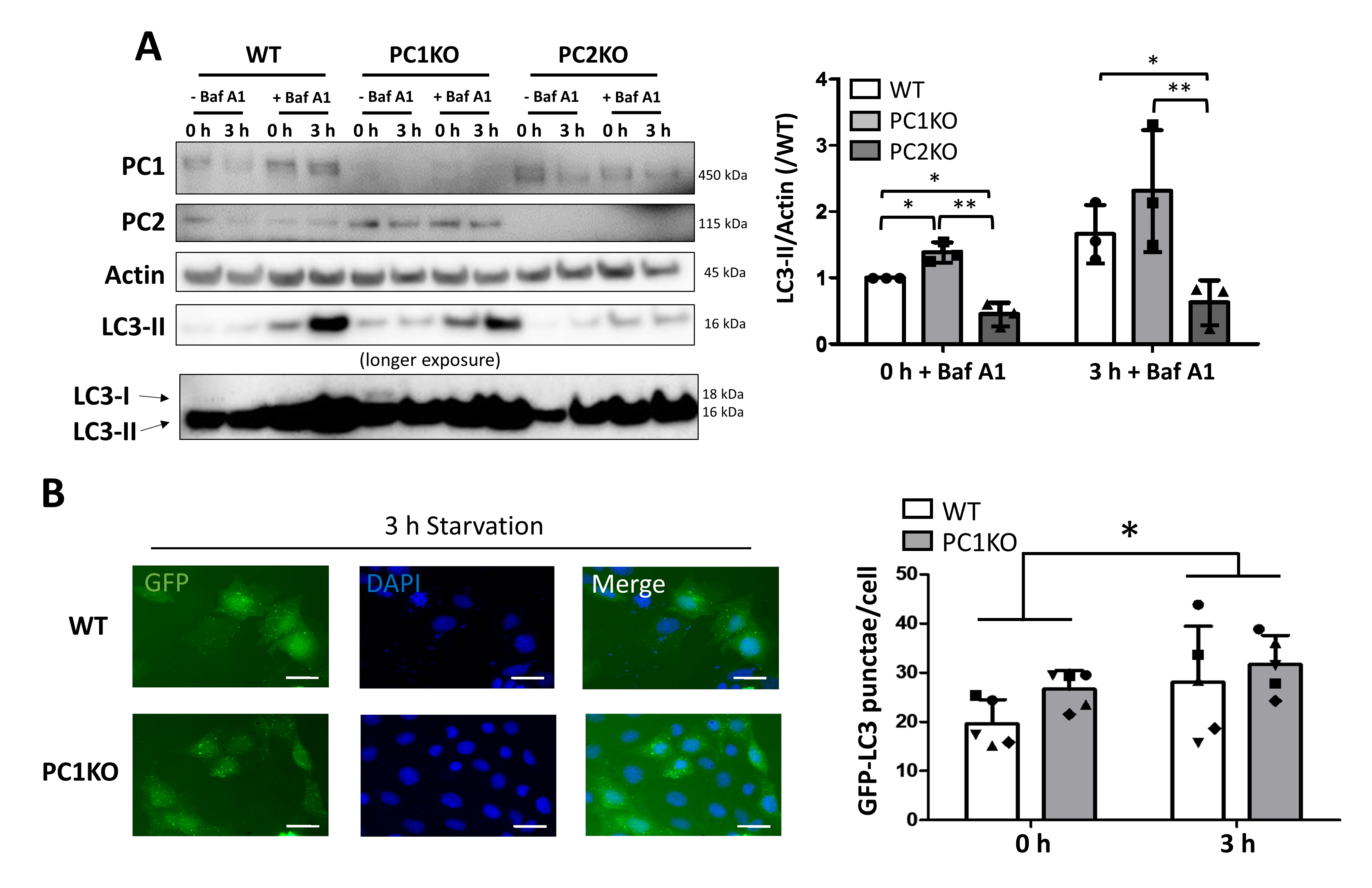

Supplement: Supplementary file 1 [file ijms-22-13511-s001.zip › New Fig. S3.tif]

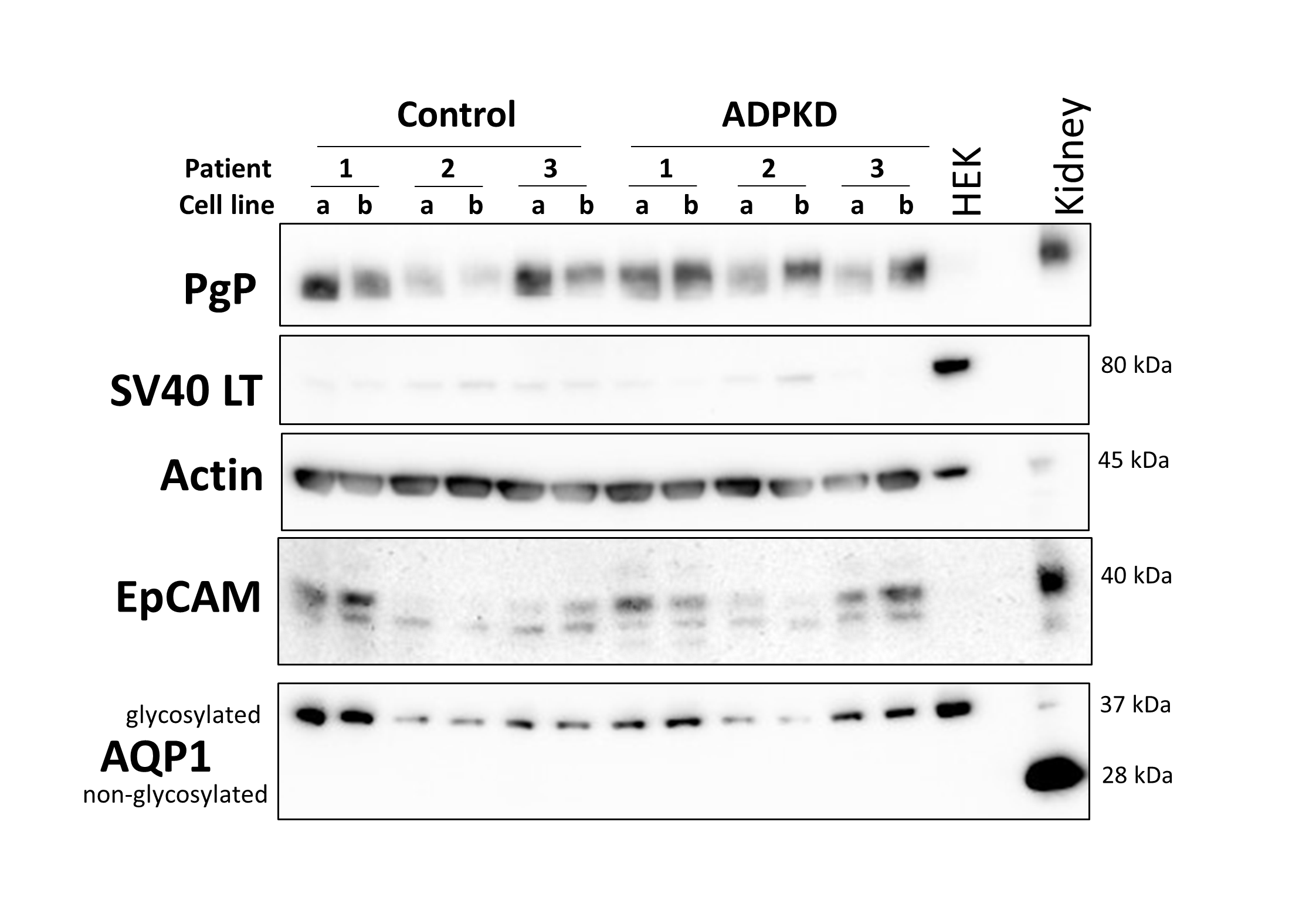

Supplement: Supplementary file 1 [file ijms-22-13511-s001.zip › New Fig. S4.tif]
